# Association of GLIM Defined Malnutrition According to Preoperative Chronic Inflammation with Long-Term Prognosis after Gastrectomy in Patients with Advanced Gastric Cancer

**DOI:** 10.3390/jcm12041579

**Published:** 2023-02-16

**Authors:** Ryota Matsui, Noriyuki Inaki, Toshikatsu Tsuji, Tetsu Fukunaga

**Affiliations:** 1 Department of Gastroenterological Surgery, Ishikawa Prefectural Central Hospital, 2-1 Kuratsuki-higashi, Kanazawa 920-8530, Japan; 2 Department of Upper Gastrointestinal Surgery, Juntendo University Hospital, 3-1-3 Hongo, Tokyo 113-8431, Japan; 3 Department of Gastrointestinal Surgery/Breast Surgery, Graduate School of Medical Science, Kanazawa University, 13-1 Takara-machi, Kanazawa 920-8641, Japan; 4 Department of Gastroenterological Surgery, The Cancer Institute Hospital of Japanese Foundation for Cancer Research, 3-8-31 Ariake, Tokyo 135-8550, Japan

**Keywords:** inflammation, gastric cancer, GLIM, malnutrition, overall survival

## Abstract

This study aimed to investigate the association of malnutrition, defined by the Global Leadership Initiative on Malnutrition (GLIM) according to preoperative chronic inflammation with long-term prognosis after gastrectomy in patients with advanced gastric cancer. We included patients with primary stage I-III gastric cancer who underwent gastrectomy between April 2008 and June 2018. Patients were categorized as normal, moderate malnutrition, and severe malnutrition. Preoperative chronic inflammation was defined as a C-reactive protein level of >0.5 mg/dL. The primary endpoint was overall survival (OS), compared between the inflammation and non-inflammation groups. Among the 457 patients, 74 (16.2%) and 383 (83.8%) were included in the inflammation and non-inflammation groups, respectively. The prevalence of malnutrition was similar in both groups (*p* = 0.208). Multivariate analyses for OS showed that moderate malnutrition (hazard ratios: 1.749, 95% concordance interval: 1.037–2.949, *p* = 0.036) and severe malnutrition (hazard ratios: 1.971, 95% CI: 1.130–3.439, *p* = 0.017) were poor prognostic factors in the non-inflammation group, but malnutrition was not a prognostic factor in the inflammation group. In conclusion, preoperative malnutrition was a poor prognostic factor in patients without inflammation, but it was not a prognostic factor in patients with inflammation.

## 1. Introduction

In 2018, the Global Leadership Initiative on Malnutrition (GLIM) criteria were published as a global agreement on malnutrition standards [1]. Malnutrition increases postoperative complications, and the risk of adverse long-term outcomes increases with the severity of undernutrition [2,3]. However, screening methods for undernutrition and preoperative nutritional interventions differ across countries and institutions. As a result, standardizing and generalizing screening for malnutrition has been difficult. The prognostic validity of the GLIM diagnostic criteria by global consensus would benefit from universal evaluation.

Malnutrition, as defined by the GLIM, is linked to a poor long-term prognosis following gastric cancer surgery [4,5], but it is unclear whether malnutrition with chronic inflammation affects prognostic outcomes in gastric cancer patients. Etiologic criteria defined GLIM criteria include not only acute disease but also chronic disease-related inflammation, such as cachexia. According to a systematic review, the neutrophil-to-lymphocyte ratio and the Glasgow prognostic score (GPS), both of which reflect inflammation, are related to prognosis [6,7]. The consensus statement of the American Society for Parenteral and Enteral Nutrition (ASPEN) recommends determining the presence of inflammation after a diagnosis of malnutrition [8]. The GLIM criteria state that C-reactive protein (CRP) can be used as a supportive laboratory measure of chronic inflammation [1]. However, it is not clear whether the clinical consequences of malnutrition differ in the presence or absence of preoperative chronic inflammation as defined by CRP.

This study aimed to investigate the association of GLIM-defined malnutrition according to preoperative chronic inflammation with long-term prognosis in patients with advanced gastric cancer after gastrectomy. We hypothesized that malnutrition would be associated with a worse long-term prognosis in patients with or without inflammation.

## 2. Materials and Methods

### 2.1. Patient Selection

This single-institution, retrospective cohort study was conducted between April 2008 and June 2018 and included consecutive patients who underwent gastrectomy for primary stage I-III gastric cancer diagnosed using the 15th edition of the Japanese Classification of Gastric Carcinoma. The inclusion criteria were (1) a primary advanced gastric cancer and (2) a gastrectomy. Patients with residual gastric cancer and cancers of other organs were excluded, as were those who underwent non-gastrectomy surgical procedures, those diagnosed with p-stage IV, and those who received preoperative treatment. This study followed the Declaration of Helsinki guidelines, and all procedures involving human subjects/patients were approved by the Institutional Ethical Review Committee of Ishikawa Prefectural Central Hospital (authorization number: 1886). All subjects/patients provided written informed consent.

### 2.2. Definition of Malnutrition and Chronic Inflammation

Before surgery, we measured the height and weight of each patient to calculate their body mass index (BMI). The graphical analysis software Ziostation was used to measure visceral fat area and skeletal muscle mass on plain computed tomography (CT) images (ZIOSOFT, Tokyo, Japan). The umbilical level was used to measure the visceral fat area (VFA), while the third lumbar vertebra level was used to measure skeletal muscle mass. Skeletal muscle mass index (SMI) was calculated by dividing skeletal muscle mass from a single CT image slice by the patient’s height in meters. Because SMI is not one of the GLIM criteria, its impact on prognosis was investigated separately. Different cut-off values for SMI were set for men and women using those previously reported for Asians [4].

The severity grades according to the phenotypic criteria used in this study are shown in Table 1. Malnutrition and its severity were determined using BMI, body weight loss (BWL) rate, and SMI in at-risk patients identified during nutritional screening using Subjective Global Assessment (SGA). According to their values, patients were classified as moderate malnutrition or severe malnutrition. Patients who did not have malnutrition were considered normal.

Preoperative chronic inflammation was defined as a CRP level of >0.5 mg/dL at the initial visit and immediately before surgery, despite the absence of acute inflammation. Patients with chronic inflammation were included in the inflammation group, while those without chronic inflammation were included in the non-inflammation group.

### 2.3. Postoperative Adjuvant Chemotherapy

In p-stage II-III, S-1 was administered as postoperative adjuvant chemotherapy at a dose of 80–120 mg/m^2^/day, with dose reduction if adverse effects were observed. Treatment was continued for up to 1 year, and no further treatment was given until recurrence. Patients who relapsed were treated according to the Treatment Guidelines of the Japanese Society of Gastric Cancer.

### 2.4. Endpoints

The primary endpoint measured was overall survival (OS), defined as the period between surgery and death. We divided the patients into two groups—those with and those without preoperative chronic inflammation—and compared the OS between patients with and without chronic inflammation in the normal and malnutrition groups.

We used the Mann–Whitney U test for continuous variables, the chi-square test or Fisher’s exact test for categorical variables, and the log-rank test for Kaplan–Meier survival analysis for OS. Multivariate analysis was performed using a forward stepwise procedure of Cox proportional hazards regression to identify prognostic factors and calculate hazard ratios (HRs). All factors indicated in the patient background were used in the multivariate analysis. The EZR software was used for all statistical analyses. The statistical significance level was set at *p* < 0.05.

## 3. Results

### 3.1. Patient Background

Table 2 shows the characteristics of included patients. Among the eligible patients, 457 were selected—74 (16.2%) and 383 (83.8%) patients were included in the inflammation and non-inflammation groups, respectively. Patients in the inflammation group were older (*p* = 0.001), had undergone more open surgeries (*p* = 0.001), had undergone more total gastrectomies (*p* = 0.034), had a high incidence of postoperative complications (*p* = 0.002), and had received less postoperative chemotherapy (*p* = 0.036) compared to those in the non-inflammation group. The prevalence of GLIM malnutrition did not differ between the groups (*p* = 0.208). The Geriatric Nutritional Risk Index and the Prognostic Nutritional Index were significantly lower in the inflammation group (*p* < 0.001, *p* < 0.001, respectively).

### 3.2. Overall Survival in All Patients

**Figure 1:**
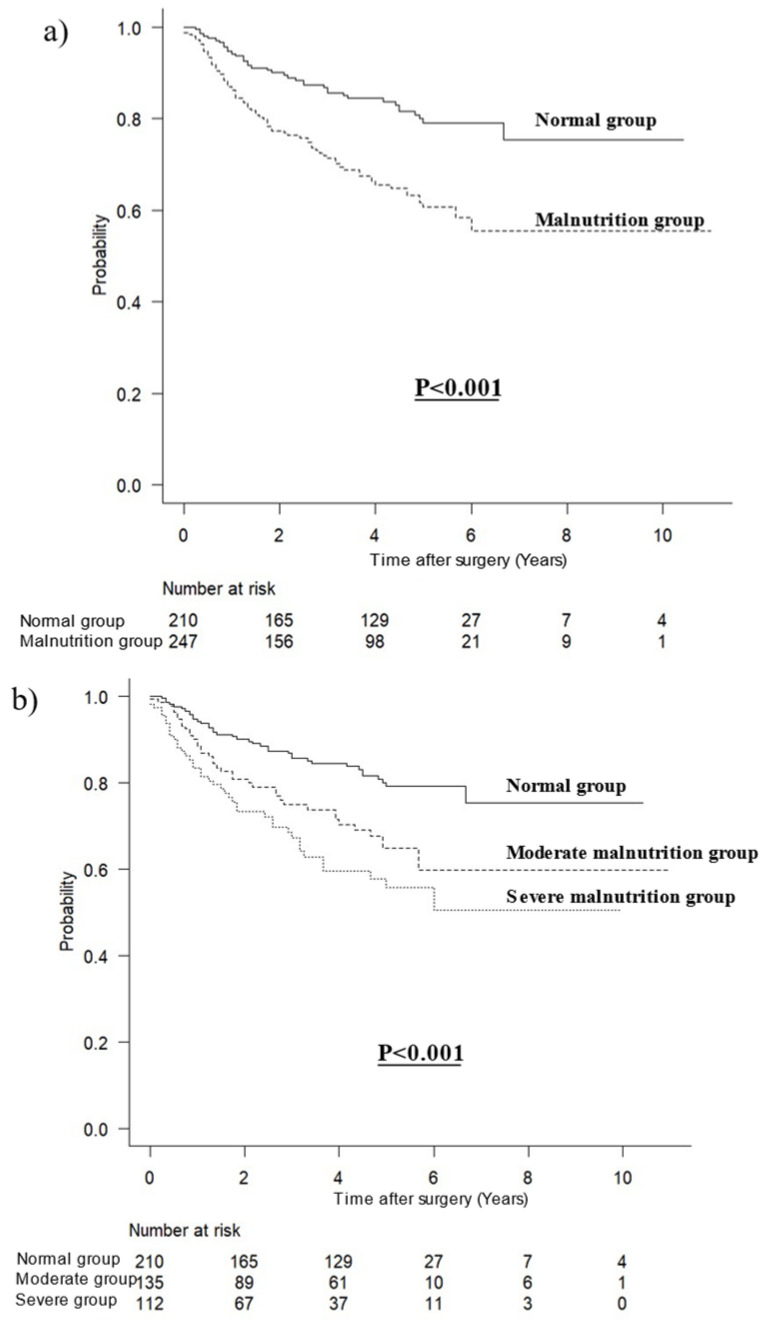

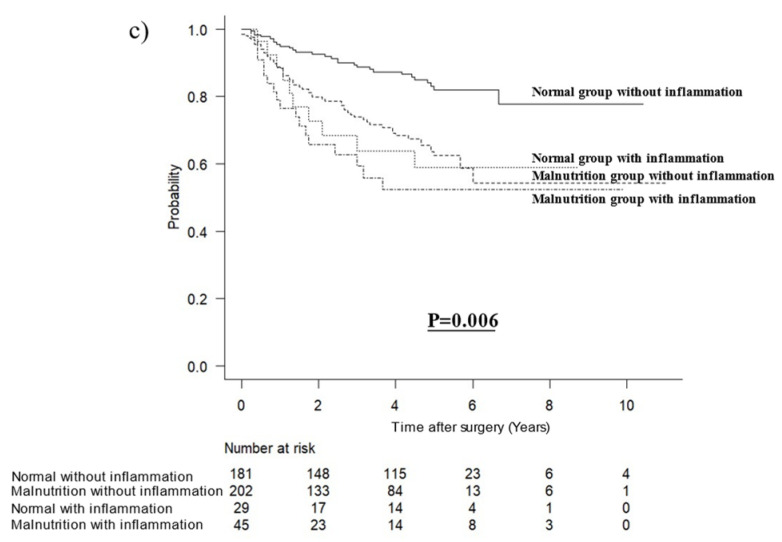
Survival curves for overall survival in all patients. (**a**) According to malnutrition, (**b**) according to severity of malnutrition, (**c**) stratified by malnutrition and chronic inflammation.

A median follow-up time of 47 months was observed (interquartile range: 19–60 months). Survival curves for OS including all patients are shown in Figure 1. The prognosis in patients with malnutrition was significantly worse than that in patients without malnutrition (*p* < 0.001, Figure 1a). The more severe the malnutrition, the worse the prognosis (*p* < 0.001, Figure 1b). According to malnutrition and chronic inflammation, the prognosis was best in the normal group without chronic inflammation (*p* = 0.006, Figure 1c).

### 3.3. Overall Survival According to Malnutrition by Inflammation

**Figure 2:**
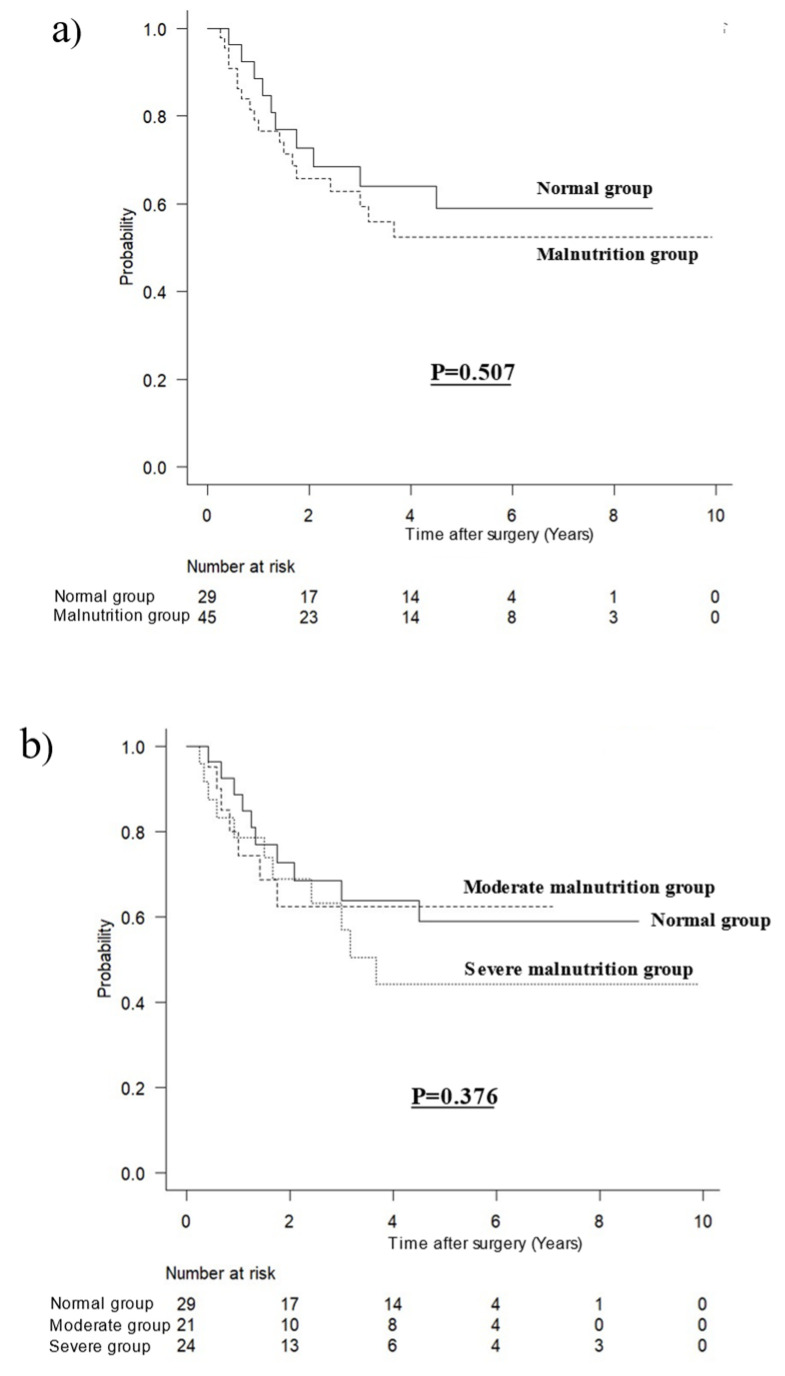
Survival curves for overall survival in patients with inflammation. (**a**) According to malnutrition, (**b**) according to severity of malnutrition.

Survival curves for OS in patients with inflammation according to malnutrition are shown in Figure 2. There was no difference in OS between patients with inflammation with and without malnutrition (*p* = 0.507, Figure 2a) and according to the varying severities of malnutrition and normal patients (*p* = 0.376, Figure 2b).

**Figure 3:**
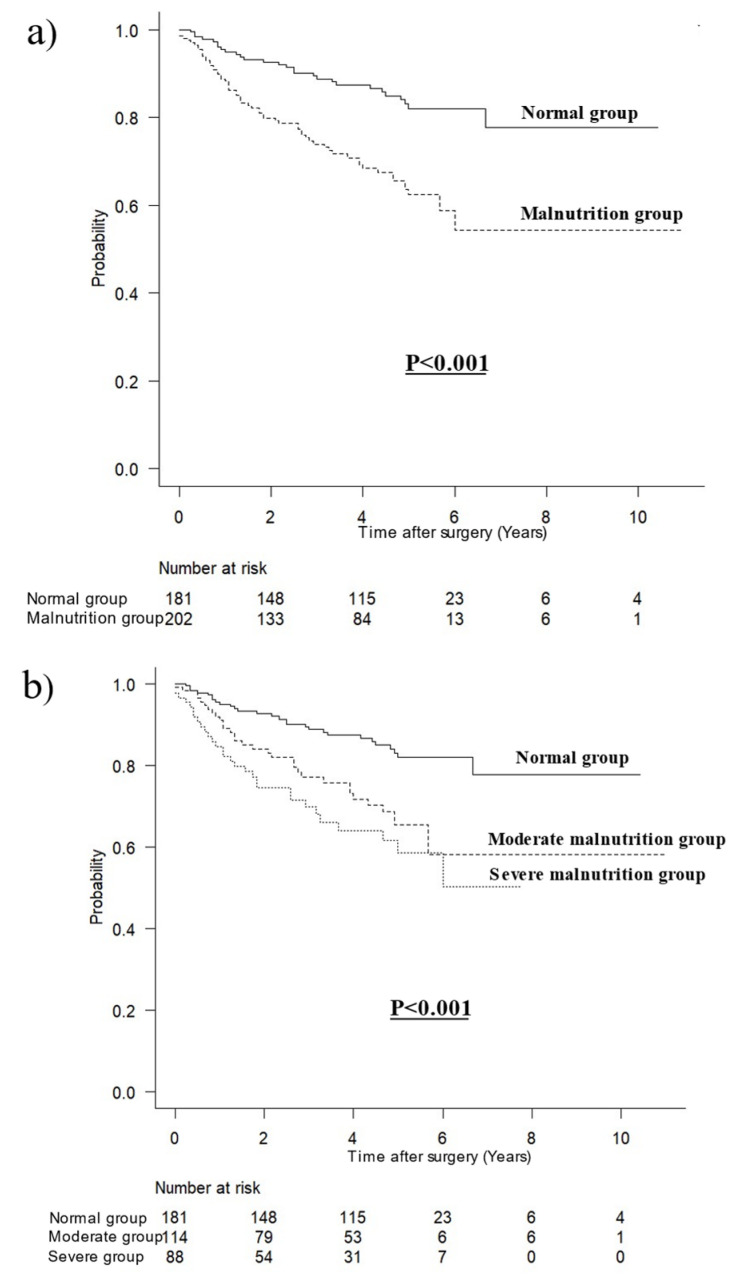
Survival curves for overall survival in patients without inflammation. (**a**) According to malnutrition, (**b**) according to severity of malnutrition.

The survival curves for OS in patients without inflammation according to malnutrition are shown in Figure 3. The prognosis in patients without inflammation was significantly worse in the malnutrition group than that in the normal group (*p* < 0.001, Figure 3a). The prognosis in patients without inflammation was significantly worse in the moderate and severe malnutrition groups than that in patients in the normal group (*p* < 0.001, Figure 3b).

### 3.4. Prognostic Factors for Overall Survival

In Table 3, the prognostic factors for OS in patients with inflammation analyzed with multivariate analysis are presented. Multivariate analysis showed that age ≥70 years (HR: 3.530, 95% confidence interval [CI]: 1.337–9.318, *p* = 0.011), serosal invasion (HR: 3.120, 95% CI: 1.191–8.173, *p* = 0.021), N3 lymph node metastasis (HR: 4.124, 95% CI: 1.815–9.370, *p* < 0.001), and undifferentiated carcinoma (HR: 0.422, 95% CI: 0.188–0.945, *p* = 0.036) were prognostic factors in patients with inflammation. Malnutrition was not a prognostic factor.

Table 4 shows the results of a multivariate analysis of the prognostic factors for OS in patients without inflammation. Multivariate analysis showed that age ≥70 years (HR: 1.956, 95% CI: 1.263–3.029, *p* = 0.003), open surgery (HR: 1.927, 95% CI: 1.229–3.021, *p* = 0.004), N3 lymph node metastasis (HR: 2.767, 95% CI: 1.765–4.338, *p* < 0.001), moderate malnutrition (HR: 1.749, 95% CI: 1.037–2.949, *p* = 0.036), severe malnutrition (HR: 1.971, 95% CI: 1.130–3.439, *p* = 0.017), VFA ≥ 100 cm^2^ (HR: 0.531, 95% CI: 0.326–0.866, *p* = 0.011), and severe postoperative complications (HR: 2.627, 95% CI: 1.474–4.681, *p* = 0.001) were prognostic factors in patients without inflammation.

## 4. Discussion

This study revealed that moderate and severe malnutrition were associated with poor long-term prognosis after gastrectomy in gastric cancer patients without inflammation; however, there was no difference in prognosis between patients with inflammation. The prognosis in patients with malnutrition was significantly worse than that in patients without malnutrition. Multivariate analyses showed that moderate and severe malnutrition were independent prognostic factors for OS in patients without inflammation but not in patients with inflammation. To the best of our knowledge, this is the first study to demonstrate the prognostic value of GLIM-defined malnutrition in patients with gastric cancer according to inflammation status.

Preoperative malnutrition was an independent poor prognostic factor in patients without chronic inflammation but not in those with chronic inflammation. Undernutrition is an important factor associated with prognosis, and it has been reported that GLIM malnutrition is a poor prognostic factor [4,5]. In this study, there was no difference in the prevalence of malnutrition between the inflammation and non-inflammation groups. The consensus statement of the ASPEN recommends determining the presence of inflammation after a diagnosis of malnutrition [8]. Patients with acute inflammation, such as preoperative pneumonia, were excluded from this study, and the inflammation group might have included patients with chronic inflammation, especially preoperative cachexia. A randomized controlled trial has shown that conventional nutritional support in the presence of inflammation does not improve prognosis [9]. In contrast, Shirai et al. showed that n-3 immunomodulatory nutritional supplements improved the prognosis of patients with cachexia and chronic inflammation [10]. Therefore, strategies to suppress inflammation, such as immunomodulatory nutritional supplements, may be effective in patients with preoperative chronic inflammation.

The other prognostic factors were perioperative factors causing inflammation, such as open surgery and severe postoperative complications. Systematic reviews have shown that the presence of inflammation is associated with poor long-term prognosis [11,12]. Chronic inflammation promotes the growth of cancer. Furthermore, chronic inflammation can promote cell malignancy and carcinogenesis [13]. Several inflammatory mediators, such as tumor necrosis factor-alpha, interleukin (IL)-6, and IL-10, have been linked to cancer initiation and progression [14]. Postoperative complications are a leading cause of inflammation and have been shown in meta-analyses to have a poor prognosis after gastrectomy [15].

Furthermore, factors related to nutritional status, such as high visceral fat levels, are associated with prognosis. In a previous study, low preoperative visceral fat mass was associated with poor prognosis [16,17]. Visceral fat presumably protects against loss of muscle mass when in the presence of chronic postoperative weight loss; however, low visceral fat mass is associated with low energy storage and poor prognosis. Therefore, strategies to detect preoperative malnutrition and provide nutritional intervention in the perioperative period may be necessary.

Malnutrition was not an independent poor prognostic factor in patients with chronic inflammation, but age > 70 years, serosal invasion, N3 lymph node metastasis, and histological type were identified as poor prognostic factors. There is no dispute that the older and more advanced the cancer, the poorer the prognosis. With regard to histological type, it has been shown that the undifferentiated type has a poorer prognosis [18,19]. In this study, patients with differentiated cancer had a higher proportion of elderly patients aged 70 years or older than those with undifferentiated cancer, which may have affected the prognosis.

The GLIM criteria are a valid diagnostic tool to determine the severity of malnutrition with phenotypic criteria for BMI, BWL, and muscle mass. We can use CT to measure muscle mass and visceral fat mass in addition to cancer staging. SMI is widely recognized as an indicator of skeletal muscle mass, and its measurement at L3 is widely used as a prognostic indicator [20]. The cut-off values for SMI in this study were based on widely used reference values in Asia. In addition, it does not require additional preoperative testing and can be widely disseminated.

This study has several limitations. First, this was a single-center, retrospective, cohort study. Second, the sample size of the inflammation group is relatively small. Third, the cut-off values for CRP are unclear and require validation in additional multicenter cohort studies. Fourth, we did not use dietary intake to make a diagnosis of malnutrition because electronic medical records contain inaccurate dietary intake data. Thus, the prevalence of malnutrition may have been higher than estimated. Fifth, the causes affecting the relationship between malnutrition and long-term prognosis in the presence or absence of inflammation are unknown. Further research, including basic research, is needed. To the best of our knowledge, this is the first study to show that malnutrition, defined by BMI, BWL, and reduced muscle mass, has an impact on overall survival after gastrectomy in patients with advanced gastric cancer who exhibit postoperative body weight loss with or without chronic inflammation. Our findings imply that there is a high need for perioperative conventional nutritional support in patients with malnutrition without chronic inflammation and perioperative immunomodulatory nutritional support in patients with chronic inflammation. As a next step, we would like to investigate whether nutritional therapy would improve OS in such patients.

## 5. Conclusions

Preoperative malnutrition, as defined by GLIM criteria, was a poor prognostic factor in patients without inflammation, but it was not a prognostic factor in patients with inflammation. This study suggested that the significance of nutritional intervention may differ depending on the presence or absence of preoperative inflammation.

## Figures and Tables

**Table 1:** Severity grade as defined by the GLIM criteria in the present study.

Severity Grade	Phenotypic Criteria		
	Weight Loss Before Surgery (%)	Low-Body Mass Index (kg/m^2^)	Reduced Muscle Mass (SMI)
Moderate malnutrition	5–10% within the past 6 months, or 10–20% beyond 6 months	<20.0 if <70 years old, or <22.0 if ≥70 years old	Male: 40.8 cm^2^/m^2^ Female: 34.9 cm^2^/m^2^
Severe malnutrition	>10% within the past 6 months, or >20% beyond 6 months	<18.5 if <70 years old, or <20.0 if ≥70 years old	Male: 34.5 cm^2^/m^2^ Female: 28.9 cm^2^/m^2^

**Table 2:** Patient characteristics.

		Total	Non-Inflammation Group	Inflammation Group	*p* Value
		(N = 457)	(*n* = 383)	(*n* = 74)	
Age, mean ± SD		67.88 ± 11.00	67.12 ± 10.98	71.82 ± 10.30	0.001
Sex	Male	301 (65.9%)	252 (65.8%)	49 (66.2%)	1
	Female	156 (34.1%)	131 (34.2%)	25 (33.8%)	
Body mass index, mean ± SD		22.90 ± 3.52	22.92 ± 3.45	22.79 ± 3.91	0.782
Surgical approach					
	Laparoscopic	253 (55.4%)	226 (59.0%)	27 (36.5%)	0.001
	Open	204 (44.6%)	157 (41.0%)	47 (63.5%)	
Performed procedure					
	Distal gastrectomy	255 (55.8%)	223 (58.2%)	32 (43.2%)	0.034
	Proximal gastrectomy	24 (5.3%)	21 (5.5%)	3 (4.1%)	
	Total gastrectomy	178 (38.9%)	139 (36.3%)	39 (52.7%)	
Lymph node dissection					0.158
	D1+	198 (43.3%)	160 (41.8%)	38 (51.4%)	
	D2	259 (56.7%)	223 (58.2%)	36 (48.6%)	
Clinical stage	I	87 (19.0%)	74 (19.3%)	13 (17.6%)	0.72
	II	72 (15.8%)	58 (15.1%)	14 (18.9%)	
	III	298 (65.2%)	251 (65.5%)	47 (63.5%)	
Pathological stage	I	88 (19.3%)	75 (19.6%)	13 (17.6%)	0.067
	II	176 (38.5%)	155 (40.5%)	21 (28.4%)	
	III	193 (42.2%)	153 (39.9%)	40 (54.1%)	
Serosal invasion	Absent	347 (75.9%)	294 (76.8%)	53 (71.6%)	0.373
	Present	110 (24.1%)	89 (23.2%)	21 (28.4%)	
Lymph node metastasis	Absent	132 (28.9%)	107 (27.9%)	25 (33.8%)	0.328
	N1	116 (25.4%)	104 (27.2%)	12 (16.2%)	
	N2	100 (21.9%)	82 (21.4%)	18 (24.3%)	
	N3	109 (23.9%)	90 (23.5%)	19 (25.7%)	
Histological type					1
	Differentiated	199 (43.5%)	167 (43.6%)	32 (43.2%)	
	Undifferentiated	258 (56.5%)	216 (56.4%)	42 (56.8%)	
Comorbidity	CKD	80 (17.5%)	65 (17.0%)	15 (20.3%)	0.505
	COPD	99 (21.7%)	77 (20.1%)	22 (29.7%)	0.089
	Diabetes	84 (18.4%)	70 (18.3%)	14 (18.9%)	0.871
	CHF	24 (5.3%)	20 (5.2%)	4 (5.4%)	1
GLIM malnutrition	Normal	210 (46.0%)	181 (47.3%)	29 (39.2%)	0.208
	Moderate	135 (29.5%)	114 (29.8%)	21 (28.4%)	
	Severe	112 (24.5%)	88 (23.0%)	24 (32.4%)	
Geriatric Nutritional Risk Index		103.9	104.7	96.85	<0.001
		(96.45–111.7)	(98.00–112.4)	(89.08–104.7)	
Prognostic Nutritional Index		49.26	49.78	43.27	<0.001
		(44.89–53.31)	(46.20–53.96)	(39.55–47.49)	
SMI (cm^2^/m^2^), median (IQR)		39.25 (34.25−45.39)	39.17 (34.47−45.67)	39.59 (32.64−44.11)	0.209
VFA (cm^2^), median (IQR)		85.00 (46.60−137.1)	83.02 (45.05−137.9)	86.00 (55.36−125.3)	0.607
≥100 cm^2^		181 (42.0%)	152 (42.2%)	29 (40.8%)	0.896
Postoperative complications					
Total complications		100 (21.9%)	73 (19.1%)	27 (36.5%)	0.002
Severe complications		45 (9.8%)	35 (9.1%)	10 (13.5%)	0.285
Abdominal abscess		53 (11.6%)	41 (10.7%)	12 (16.2%)	0.17
Pneumonia		16 (3.5%)	11 (2.9%)	5 (6.8%)	0.155
Incisional SSI		10 (2.2%)	8 (2.1%)	2 (2.7%)	0.668
Anastomotic leakage		21 (4.6%)	13 (3.4%)	8 (10.8%)	0.011
Pancreatic leakage		30 (6.6%)	26 (6.8%)	4 (5.4%)	0.802
Postoperative chemotherapy					
	Absent	176 (38.5%)	139 (36.3%)	37 (50.0%)	0.036
	Present	281 (61.5%)	244 (63.7%)	37 (50.0%)	

CHF chronic heart failure, CKD chronic kidney disease, COPD chronic obstructive pulmonary disease, IQR interquartile range, SD standard deviation, SSI surgical site infection.

**Table 3:** Prognostic factors for overall survival in patients with inflammation.

Variables		Multivariate Analysis		
		HR	95% CI	*p* Value
Age (years)	<70	1		
	≥70	3.53	1.337–9.318	0.011
Serosal invasion	Absent	1		
	Present	3.12	1.191–8.173	0.021
Lymph node metastasis	Absent	1		
	N3	4.124	1.815–9.370	<0.001
Histological type	Differentiated	1		
	Undifferentiated	0.422	0.188–0.945	0.036

**Table 4:** Prognostic factors for overall survival in patients without inflammation.

Variables		Multivariate Analysis		
		HR	95% CI	*p* Value
Age (years)	<70	1		
	≥70	1.956	1.263–3.029	0.003
Surgical approach	Laparoscopic	1		
	Open	1.927	1.229–3.021	0.004
Lymph node metastasis	Absent	1		
	N3	2.767	1.765–4.338	<0.001
GLIM malnutrition	Normal	1		
	Moderate	1.749	1.037–2.949	0.036
	Severe	1.971	1.130–3.439	0.017
Postoperative complication	Absent	1		
	Severe complications	2.627	1.474–4.681	0.001
VFA (cm^2^)	<100	1		
	≥100	0.531	0.326–0.866	0.011

## Data Availability

The datasets generated and/or analyzed during the current study are available upon reasonable request from the corresponding author.
